# Emerging Technologies for In Vitro Inhalation Toxicology

**DOI:** 10.1002/adhm.202100633

**Published:** 2021-07-22

**Authors:** Ajay Vikram Singh, Anthony Romeo, Kassandra Scott, Sandra Wagener, Lars Leibrock, Peter Laux, Andreas Luch, Pranali Kerkar, Shidin Balakrishnan, Sarada Prasad Dakua, Byung‐Wook Park

**Affiliations:** ^1^ Department of Chemical and Product Safety German Federal Institute for Risk Assessment (BfR) Max‐Dohrn‐Strasse 8‐10 Berlin 10589 Germany; ^2^ Department of Chemical Engineering Rayen School of Engineering Youngstown State University Youngstown OH 44555 USA; ^3^ ICMR – National AIDS Research Institute (NARI) Pune Maharashtra 411026 India; ^4^ Department of Surgery Hamad Medical Corporation (HMC) PO Box 3050 Doha Qatar

**Keywords:** air–liquid‐interfaces, inhalation, lungs‐on‐chip, machine learning, toxicology

## Abstract

Respiratory toxicology remains a major research area in the 21st century since current scenario of airborne viral infection transmission and pollutant inhalation is expected to raise the annual morbidity beyond 2 million. Clinical and epidemiological research connecting human exposure to air contaminants to understand adverse pulmonary health outcomes is, therefore, an immediate subject of human health assessment. Important observations in defining systemic effects of environmental contaminants on inhalation metabolic dysfunction, liver health, and gastrointestinal tract have been well explored with in vivo models. In this review, a framework is provided, a paradigm is established about inhalation toxicity testing in vitro, and a brief overview of breathing Lungs‐on‐Chip (LoC) as design concepts is given. The optimized bioengineering approaches and microfluidics with their fundamental pros, and cons are presented. There are different strategies that researchers apply to inhalation toxicity studies to assess a variety of inhalable substances and relevant LoC approaches. A case study from published literature and frame arguments about reproducibility as well as in vitro/in vivo correlations are discussed. Finally, the opportunities and challenges in soft robotics, systems inhalation toxicology approach integrating bioengineering, machine learning, and artificial intelligence to address a multitude model for future toxicology are discussed.

## Graphical abstract

The development of breathing human lungs‐on‐chip brought about the advent of disrupting experimental strategies in inhalation toxicology. In this review, the recent development is explored in the field and future recommendations are provided to advance the in vitro inhalation toxicology integrating emerging air–liquid interface design, innovative soft robotics, and bioMEMS technologies.

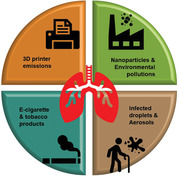

## 1 The Lung as the Target Organ for the Emission from Emerging Technologies and Engineered Nanomaterials (ENMs)

**Figure 1:**
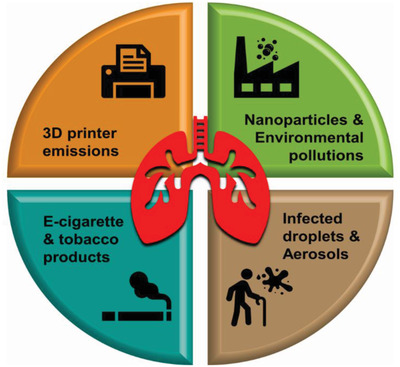
Respiratory systems as a gateway to versatile inhalation. The inhaled multiscale (milli‐, micro to nanoscale) particulate includes pollutant, infectious agents, and pollutant emissions. The household 3D printers and electronic cigarettes (e‐liquids/e‐cigarettes) are known to release invisible emissions passively inhaled into the respiratory system.

Inhalation represents a major route of exposure in which individuals breathe in a variety of substances in air, including pollutants entering the respiratory tract.^[^ ^1^ ^]^ With emerging new technologies, such as 3D household printing^[^ ^2^ ^]^ and electronic cigarettes, have attracted the attention of regulators to inhalation exposure.^[^ ^3^ ^]^ In addition, in light of the current global pandemic, the inhalation toxicity model becomes even more important in the context of infectious aerosols and ENMs used in emerging technologies^[^ ^4^ ^]^ as shown in **Figure** **1**.

In terms of ENMs exposure, there is a lack of available models that can provide safety information. The current models range from simple cell line‐based monocultures, 3D systems, and organoids for physicochemical properties of ENMs.^[^ ^5^ ^]^ Some in vitro approaches replicate various ENMs interactions with respiratory line cells and microbiota. These approaches should focus on the study of engineered nanomaterials to further improve the field of nanosafety in context with inhalation toxicology.

**Figure 2:**
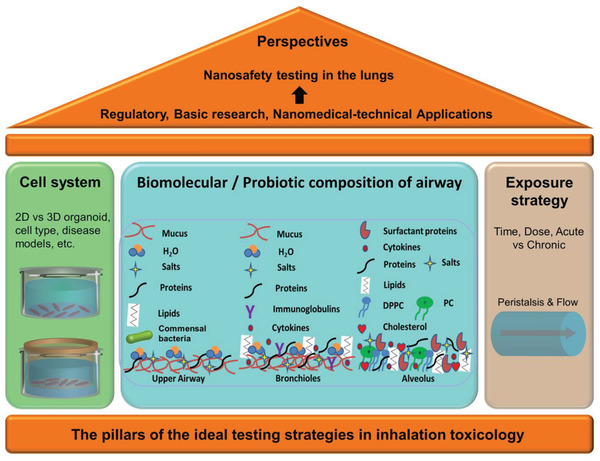
The three pillars in inhalation toxicology with scope to idealize testing strategies. Significant mammalian/human cell advances have been made (panel with left content). The conditions and strategies mimicking exposure in vivo have caught attention. The physicochemical environments in upper, middle, and lower respiratory lining such as pH, non‐aqueous component of mucus such as mucin, bimolecular composition, and salt microenvironment influence particle sedimentation. Dissolution and aggregation compromise the availability of these particulate matters on the cell surface to assess cell‐bioorganic matter interactions. Further, the versatile matter entering the inhalation pathway makes substantial contact with the microbiota in upper and lower respiratory lining for an extended period. Considering the complex physicochemical properties of these matters can create adverse effects on the microbial community and it has not been realized within in vitro toxicology (panel with middle content). Further, the exposure strategy following ALI at deep lung and dynamicity are additional key factors that need attention (panel with right content).

Particle emission from industry and vehicle combustion sources is of major concern for human health. Previous studies have indicated around 10^12^ to 10^13^ particles per day inhaled by a single person.^[^ ^6^ ^]^ This study was based on the oral intake of up to 5.4 mg day^−1^ TiO_2_ and 35 mg day^−1^ silica microparticles. In considering current numbers from the European Food Safety Authority (EFSA) the average exposure for adults was 63–189 mg day^−1^ of TiO_2_ and silica. Both Dekkers et al and the EFSA estimated 9.4 and 0.9–27 mg/kg bodyweight/day, respectively.^[^ ^7^ ^]^ Toxicological investigations with ENMs have shown that extent of negative effects relies on their physicochemical properties, including, size, agglomerate, solubility, shape, and surface reactivity. Within the respiratory tract, ENMs face complex environments (e.g., multifaceted immune systems, cell morphometry, abiotic components, etc.) and processes that can alter their physicochemical properties, because of their interactions with toxicologically relevant target cells.^[^ ^7^, ^8^ ^]^ These complexities need attention to normalize the dose metrics in accordance with the macrophages rather than the alveolar surfaces to help produce in vitro to in vivo extrapolation of the results. The advances in cell systems, mimicked exposure pathways, and exposure strategies pave the three major components (pillars) in advanced inhalation toxicology as shown in **Figure** **2**. The airway‐on‐chip platforms in this direction contributed to reliably mimic the exposure on in vitro models of respiratory inhalation.^[^ ^9^ ^]^

## 2 Particle Characteristics as a Function of Dosimetry and In Vivo Depositions‐Clearance

**Figure 3:**
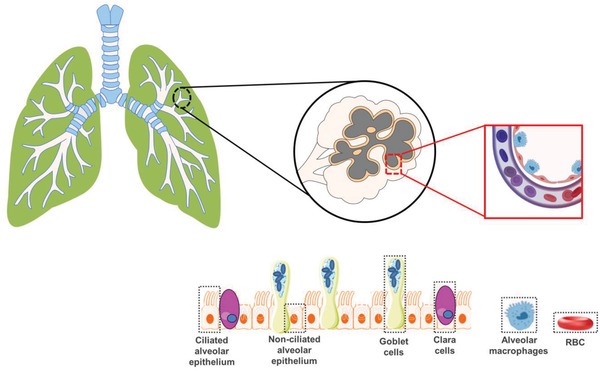
Morphometric description of versatile respiratory pathway. The extrathoracic, thoracic, tracheobronchial, and pulmonary region are established with the specialized cells of ciliated and non‐ciliated alveolar epithelium. The ALI at the lung‐circulation region has very specialized cells with distinct functional profiles such as clara, goblet cells, and alveolar macrophages (magnified region) which take part in muco‐ciliary clearance of infectious agents and multiscale particulate matter. This is worth considering while designing ALI within in vitro inhalation toxicology experiments. These epithelial and endothelial cell types originate from the alveolar stem cells. The differentiation takes place in response to dynamic air exchange and localized signaling while progenitor cells travel along the ALI. Created with Adobe Illustrator and Biorender.

**Figure** **3** provides a simplified illustration of the inside human respiratory tract after air enters the nose and/or mouth during breathing, passing through the nasopharyngeal, laryngeal or extra thoracic region of the respiratory tract.^[^ ^10^ ^]^ After passing through the conducting airways in the upper part of the lung, the air enters the deep lung where the windpipe branches repeatedly, divide into a series of airways referred to as bronchi and bronchioles (**Table** **1**). The air moves slowly as it passes deep inside the lung and finally reaches the alveolar center where it almost remains stationary in the alveoli.^[^ ^11^ ^]^ During the movement of the air, oxygen diffuses into the alveolar wall and enters the blood stream. At the same time, carbon dioxide is released from the bloodstream and exposed to the air during exhalation. If particles present in the air are inhaled, they are transferred with the air into the respiratory tract.^[^ ^12^ ^]^ Here, particle size is one of the most important factors. The smaller particles may deposit into the respiratory tract, because the smaller particles have lower inertia.^[^ ^13^ ^]^ However, most of the particles that failed to deposit in the respiratory tract sail back with the exhaled air. Detecting and monitoring the particles deposited in the respiratory tract help define the health consequences of particle exposures.^[^ ^14^ ^]^ The specific conditions can be considered: a) the particles may be cleared from the respiratory tract by alveolar macrophages or the muco‐ciliary escalator without causing harm; b) they may cause direct injury to the respiratory tract where they could be deposited or translocated to other parts of the body via blood or lymphatic circulation; c) potentially lead to damage of organs or tissue outside of the respiratory tract.

**Table 1:** Hallmark of inhalation pathway with hierarchy in structure and functions

Region anatomy	Anatomy	Cell profile	Functions
Extrathoracic	Nose (anterior larynx) and mouth (posterior pharynx)	Olfactory epithelial cells, basal cells, mucous secreting goblet cells, non‐ciliated, and ciliated epithelial cells	Air conditioning via temperature‐humidity control, particulate clearance, ciliated cells sweep debris
Thoracic	Trachea and bronchi	Non‐ciliated and ciliated epithelial cells, respiratory epithelial cells, Clara cells secretion metabolize toxicant and divide/differentiate	Clearance of medium particle sizes (10–100 nm) gas exchange and air conduction
Pulmonary	Alveolar duct/sac	Squamous (94% type 1 cells) and cuboidal alveolar epithelial cells (6% Type II)	ALI region with gaseous exchange to systemic circulation

With the inhalable sampling criterion, one can predict the percentage of particles deposited in the nasopharyngeal and laryngeal regions as a function of particle size. The International Commission on Radiological Protection suggests that roughly 20% of 10 nm particles are deposited in the nasal, pharyngeal and laryngeal region whereas only about 4% of the 300 nm particles will deposit in these regions.^[^ ^15^ ^]^ Nearly all the 1–10 micrometer particles inhaled deposit in the nasopharyngeal and laryngeal regions. A large fraction of smaller (size range) particles entering the tracheobronchial region collects on the walls of the bronchial/bronchioles due to diffusion caused by Brownian motion.^[^ ^16^ ^]^ However, some of the particles still have inertia and collect near bifurcations as larger bronchial airways split into smaller ones. The alveoli at the end of the smallest bronchioles in the lung are formed like leaves on the branches of a tree as seen in Figure 3. A healthy human adult has roughly 500 million alveoli. The surface area of all the alveoli in a human adult would be larger than a basketball court.^[^ ^17^ ^]^ Upon closer inspection, the alveoli appear in clusters. Small particles penetrating the alveolar region can diffuse into the alveoli and are deposited there. Since the residing time of air in the alveolar region is relatively long, a small portion of them can be exhaled.^[^ ^18^ ^]^

The aspiration and instillation exposure techniques as recommended by Organization for Economic Co‐operation and Development (OECD) can be used as testing guidelines to detect the local acute lung toxicity in inhalation experiments in vitro.^[^ ^19^ ^]^ For the biological situation in the lung, biological barriers separate the inner body from the outer compartment. It is a complex organ with the functional organization of different cell types, specific regional characteristics depending on biological functions, complex ventilation, and fluid dynamics together as described in Table 1.^[^ ^20^ ^]^ In modeling a system for local acute lung toxicity, the system includes only the most important characteristics of the lung, which can produce an accurate model.

### 2.1 Perspective of In Vitro Alternatives to Acute Inhalation Toxicity Studies in Animal Models

Inhalation is a prime exposure route for humans and the respiratory epithelium is the first tissue that inhaled substances come in contact with.^[^ ^21^ ^]^ To date, no in vitro models have been accepted by regulatory agencies as a complete replacement for inhalation toxicity testing in animals. In animal models, acute inhalation toxicity testing for regulatory purposes in rats or mice is done according to OECD TG403, TG436, and TG433 test guidelines.^[^ ^22^ ^]^ The differences in the respiratory tract's structural design and function between species create bias and renders it more challenging to develop similar results in humans.^[^ ^11^ ^]^ Two issues known to control the toxic effects of inhaled substances in animal models are the deposition pattern of the test substance and the routes the compound is discharged from the lungs. All animal models differ from humans in these aspects.^[^ ^23^ ^]^

The Interagency Coordinating Committee on the Validation of Alternative Methods was formed in 2000 in the US. to ascertain a precedent of alternative approaches for animal testing. They examined in vivo, in silico, and in vitro tests for acute systemic toxicity and developed a plan that speeds the substitution of current animal testing for in vitro and in silico.^[^ ^24^ ^]^ In 2016, the environmental protection agency announced the goal of ending funding for mammal testing by 2035. However, this progress has been slow. Regulatory authorities approve validation to in vitro alternative tests upon proof of their ability to predict in vivo animal‐derived data. The data's reliability is inadequate and the process takes time, in some cases up to 10 years, and can cost over a million dollars. For example, steps for validation include an endorsement from the European validation authority and testing with large international collaboration platforms, such as the OECD or the International Council on Harmonization (ICH), followed by regulatory acceptance and removal of the animal test.^[^ ^24^ ^]^

## 3 Advanced in vitro *models* Mimicking Air–Liquid Interface (ALI) in the Lungs: Miniature Micro‐Lung and Metabolic‐Lung Models

**Figure 4:**
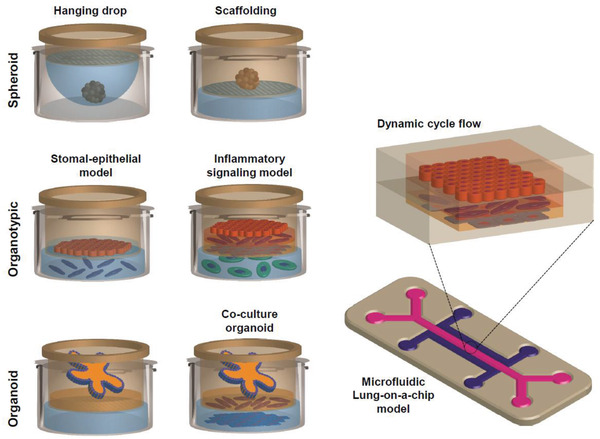
Advances in the development of in vitro models mimicking ALI in the lungs. Current Transwell‐based ALI systems adopt the most common multicellular 3D model originated from specific inflammatory cells and signaling modes adopting versatile culture conditions (left panel). Adopting suitable differentiation protocol involving scaffold materials and structures with mono‐ or co‐culture and primary cells are under current investigation, bridging the gap of interlaboratory variations. The dynamic cyclic flow and pulsatile microfluidic cultures can be utilized for LoC models mimicking lung ALI (right panel).

This section reviews the challenges of replacing the in vivo animal model with in vitro advance methods and the reasons surrounding the limited innovations.^[^ ^25^ ^]^ In traditional animal testing for inhalation toxicology, the models have used many rodents such as mice, hamsters, and guinea pigs. However, the problem is that their physiology and anatomy are widely different from humans.^[^ ^26^ ^]^ It is difficult to understand the human lung response from in vivo rodent data simply by simulating inhalation in constant feeding of nano‐/micro‐particle such as silica or air pollutant, which are necessary for acute or chronic time points.^[^ ^7^ ^]^ With histology for the lung, it is required to look for any types of inflammatory mediators that identify inhalation biomarkers. However, this approach often shows inter‐laboratory variations, which may lead to the advent of in vitro lung models developed from the cell repository or developed from human tissue banks.^[^ ^27^ ^]^ Further interlaboratory variations arise due to simulated culture differences (e.g., organoids versus spheroids), dynamic cyclic flow of medium (mechanics), and physiological behavior simulated therein as shown in **Figure** **4**. Particularly organoid system complement well the microphysiological toxicity assay since it represents complete 3D organ models, which can be tamed with environmental culture conditions to see the response against a specific toxicant or mutagens. Advances in induced pluripotent stem cells (iPSCs) made organoid culture‐based assay a favorite model in inhalation toxicology from a species‐specific point of view or toxicological perspective.

**Figure 5:**
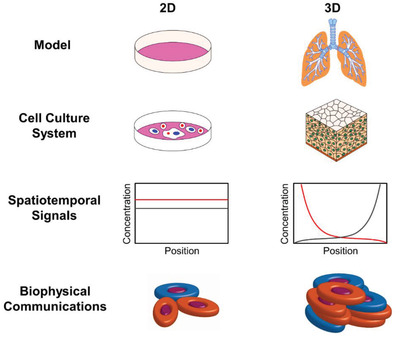
Schematic checklist describes key parameter differences in 2D versus 3D culture environment that should be considered in vitro inhalation toxicology assay. The model panel and cell culture system show that the culture substrate (3D versus 2D) adopting human‐relevant experimental exposure in third dimensions (z‐) brings in vivo lung‐like biological function, such as cell differentiation, proliferative activity, cell shape, and polarity. The molecular distribution and biophysical properties panel exhibit that altering mechanical cues in third dimensions in combination with substrate stiffness, sheer flow brings in vitro model closer to in vivo providing the correct set up of spatiotemporal molecular distribution of biochemical cues and biophysical properties. Adapted with permission.^[^ ^32^ ^]^ Copyright 2014, American Chemical Society.

To develop microlung, the normal human bronchial epithelial cells are retrieved from live donors or even post‐mortem donors from medical waste tissues and the progenitors or basal epithelial cells are removed.^[^ ^28^ ^]^ These cells are seeded with 3D differentiation kits established with specific research lab requirements. The isolated cells can be differentiated into seven different cell types like bronchial epithelium when grown in the air at liquid interphase. This microlung in Transwell mimics real breathing lungs with ciliated cells, goblet cells, and clara cells that secrete intranasal antioxidants, when exposed to irritants in the lung. There are also intermediate cells and basal cells, which interchangeably turn into each other by changing the appropriate media.^[^ ^29^ ^]^ The miniaturized micro lung system works well with in vitro inhalation exposure, tissue histology, biochemical genomics, and proteomics analysis can be performed on these microsystems routinely like in vivo animal models.^[^ ^30^, ^31^ ^]^ As shown schematically in **Figure** **5**, these miniature lung models essentially include all 3D features, which makes them unique to their 2D in vitro culture models.^[^ ^32^ ^]^

There are several advantages to using these Microlung systems, including many in vivo pseudostratified lung epithelia characteristics such as the 7 different cell types, expression of tight junctions and adherent junctions, desmosomes and hemi‐desmosomes, cilia, and microvilli.^[^ ^22^ ^]^ Researchers also achieve lung immunology when they secrete cytokines and mucin, without any cancerous transfected genes. Muco‐ciliary phenotype in vivo exists in humans resembling microscopic features visible in thin sections about 10 microns in length with the basal cells and goblet cell. Like in vivo ciliated cells, the cilia beat back and forth and spread mucin across the whole surface. The other remarkable aspect of a microlung cell culture is that it gets 9 microtubules fused outside and 2 unfused pairs of microtubules in the center of a cylindrical cilia (9 + 2 organization) like in true cilia of basal bodies under high‐resolution electron microscopy, which cannot be achieved in the submerged culture.^[^ ^33^ ^]^ The ultrastructural (9 + 2) axoneme expression is an indication of functional and motile eukaryotic flagella/cilia development on in vitro culture platform. Biochemical characteristics can resemble in vivo secretions of the glycocalyx composition with cilia, which beats synchronously back and forth with well‐developed tight junctions, adherence junctions, and desmosomes when long term culture was performed up to 33 days. The microlung and metabolic lung can be adapted to inhalation toxicology analysis to investigate all the relevant toxicological assays, biochemical and immunological screenings for short and long‐term cultures. It is also possible to measure apicobasal transepithelial/endothelial electrical resistance (TEER), fitting conventional EndOhm chamber to characterize tight versus leaky barriers upon cell injury when exposed to a toxicant in a time and dose‐dependent manner. Cell viability characterization can be accessed via quantifying ATP secretion and their TEER correlation with growth, plateau, and demise of cells on the microlung assays.^[^ ^34^ ^]^ The 3D metabolic lung can be combined with the in vitro 3D hepatocyte models to building in vivo like metabolism investigation combined with inhalation procedures. Berlin‐Brandenburg research platform^[^ ^35^ ^]^ or BBR3 advocates to avoid animal experiments altogether (**R**eplacement), to limit the number of animals (**R**eduction), and their suffering (**R**efinement), originally proposed by Russel and Burch.^[^ ^36^ ^]^ Emerging therapies for inhalation toxicology can largely benefit using these advanced human tissue‐based models of respiratory epithelium following BB3R principles of emission from 3D printers, e‐cigarettes/e‐liquids, nanoaerosols, and infectious droplets. Desired exposure of acute, chronic, sub‐chronic, and repeat window for detailed toxicological analysis at an affordable cost can be applied to develop optimized in vitro models.^[^ ^37^ ^]^

## 4 Case Reports: Breathings Lungs‐on‐Chip Model for Inhalation Toxicology

**Figure 6:**
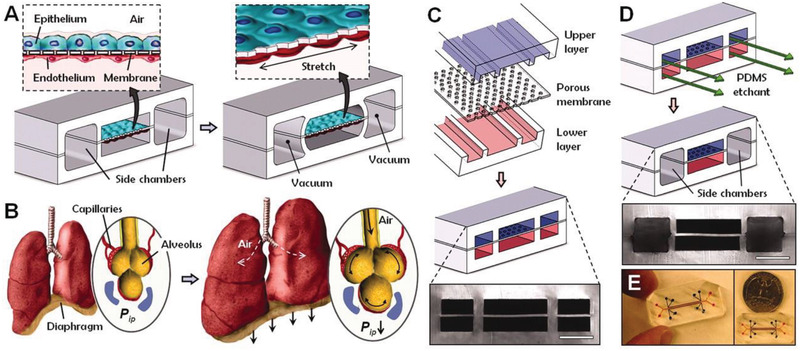
Human breathing lungs‐on‐chip technology. A) The microfabricated lung mimic device uses a compartmentalized PDMS microchannel to form an alveolar–capillary barrier on a thin, porous, flexible PDMS membrane coated with ECM. The device recreates physiological breathing movements by applying vacuum to the side chambers and causing mechanical stretching of the PDMS membrane forming the alveolar–capillary barrier. B) During inhalation in the living lung, contraction of the diaphragm causes a reduction in intrapleural pressure, leading to distension of the alveoli and physical stretching of the alveolar–capillary interface. C) Three PDMS layers are aligned and irreversibly bonded to form two sets of three parallel microchannels separated by a 10‐µm‐thick PDMS membrane containing an array of through‐holes with an effective diameter of 10 µm. D) After permanent bonding, PDMS etchant flows through the side channels. Selective etching of the membrane layers in the channels produces two large side chambers where a vacuum is applied to cause mechanical stretching. Scale bar, 200 µm. E) Images of an actual LoC microfluidic device viewed from top. Reproduced with permission.^[^ ^46^ ^]^ Copyright 2010, American Association for the Advancement of Science (AAAS).

The human Organ‐on‐Chip are micron‐sized physiological cell culture devices that mimic the essential structure and the functional units of human organs for a variety of applications.^[^ ^38^ ^]^ These models have great potential for a variety of applications in pharmaceutical, toxicology, and disease model development. The organs on chip offer a plethora of possibilities:^[^ ^39^ ^]^ a) to tame the spatiotemporal organization of tissue hierarchy and architectures that exist in vivo like ALI as shown in **Figure** **6**; b) to tame the intensity, dose, and rate of the biomechanical stimuli; c) real‐time monitoring, visualization and influence of the cell, tissue and organ functions via applying mechanical forces mimicking pulsatile organs in vivo.^[^ ^40^ ^]^

LoC revolves around developing the microfabricated devices that are used to grow human cells and mimic the complex structure and environment in the unity of human organs and body in ways that have not been possible using traditional secular models and techniques. These systems are used as micro‐engineered models of human organs to be functional units for a variety of applications for drug testing, toxicology screening, environmental monitoring, and mechanistic disease studies.^[^ ^41^ ^]^ The mobile sim card size devices called human Organ‐on‐Chip are micro‐electro‐mechanical systems (MEMS).^[^ ^42^ ^]^ In this section, we will introduce this technology using a few specific human organ and chip models that were developed exclusively in context with inhalation toxicology.^[^ ^43^ ^]^ The LoC are microfluidic devices designed to mimic the human lungs. In vivo whole lung is a complex organ consisting of functional units called alveoli that are microscopic air sacs deep in the lung, which expand and contract during respiration. These air sacs are covered with pulmonary capillaries with tunable gas exchange and the interface between an alveolus and systemic circulation.^[^ ^44^ ^]^ Surrounding capillaries are composed of lung tissue with alveolar epithelial tissue and capillary tissue on respective sides separated by the very thin interstitial membrane of few micrometers. To mimic this structure and the dynamic environment of the alveolar system, engineers used micro fabrication techniques to build a device called human breathing along on a chip.^[^ ^45^ ^]^

Figure 6 shows the entire device made of transparent and biocompatible silicone elastomer called PDMS or polydimethylsiloxane. View from the cross‐section of the device, there are upper (respiratory side) and lower (circulation side) cell culture chambers separated by a thin porous flexible membrane mimicking ALI.^[^ ^47^ ^]^ The lung cells can be cultured on one side and capillary cells on the other side to mimic the original structure of the alveolar–capillary interface. To create dynamic motion, the cyclic vacuum suction is applied inside the side chambers to stretch the tissue layers to mimic cyclic breathing motions in the microfluidic device. Emerging technologies with lung simulation will greatly assist in the future to develop such synthetic micro/multiscale organ systems for inhalation toxicology of chemicals and ENMs.^[^ ^50^, ^51^, ^52^, ^53^ ^]^ On these platforms, authors demonstrated that silica NPs exposed to alveolar epithelium grown simulating ALI under mechanical stretching shows upregulation of ICAM‐1 (Intercellular Adhesion Molecule 1). Biochemical profiling demonstrated that alveolar cells reduce ROS, and these effects were present only when both the condition (NPs + strain) exist together. Changing materials type from silica to polystyrene nanoparticles or superparamagnetic nanoparticles exhibited increased cellular uptake or transient ROS production respectively.

**Figure 7:**
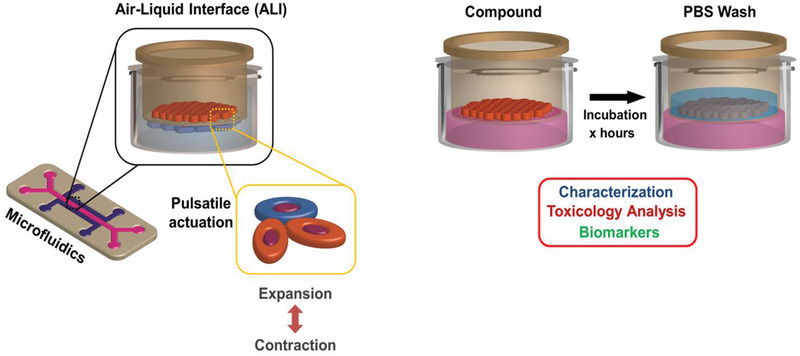
Essential components of in vitro organs‐on‐chip. Integrating on chip mechanical stretching of a functional ALI of alveolar–capillary junction (left panel) assures long‐term perfusion culture establishing tissue‐tissue interfaces and bimolecular mimicry (e.g., surfactant production). It also establishes TEER‐like in vivo tight junction protein expressions induced by membrane stretching under applied pulsatile tension. This opens a plethora of opportunities in acute and chronic in vitro inhalation toxicology measure. There are innumerable toxicity‐related novel biomarkers identification/endpoints, assay validation for efficacy/toxicity testing under varying media flow, incubation and culture manipulation (right panel).

The majority of the Organ‐on‐Chip systems (e.g., lungs, kidney, heart, liver, etc.) need microfluidic‐based continuous cell media perfusion. However, only the lungs and heart require pulsatile mechanical stimuli. Generally, the Organ‐on‐Chip systems utilize laminar, pulsatile, or interstitial flow regimes produced via microfluidics tamed devices.^[^ ^48^ ^]^ The dynamicity is achieved via strain (stretch) or compression forces created by pneumatic pumps or equivalent devices along with injecting cell cultures media in microfluidic chambers grown with appropriate cell systems as shown in **Figure** **7** below. The flow or medium perfusions can also be obtained by a gravity‐driven passive leveling. This device allows the culturing of human cells in a physiological environment: air on the lung side and blood flow on the capillary side. It eventually forms a living bilayer tissue that looks like the structural unit of the lung.^[^ ^49^ ^]^ **Table** **2** shows several advance perfusion systems used in commercial ´Organs‐on‐Chip´ modules to mimic in vivo microphysiology. These perfusion systems can significantly support inhalation toxicology in vitro.

**Table 2:** Commercial Organ‐on‐Chip technologies with dynamic flow and 3D perfusions culture capabilities, which can be potentially customized into LoC for inhalation toxicology

Organ‐on‐Chip technology	Commercial manufacturer	Remarks	URL
Microfluidic chip	Fluigent	Microfluidic solution to organs on chip technology	https://www.fluigent.com/
Rotary Cell Culture System	Synthecon	3D Biological scaffolds (Biomerix & BioFELT) technology amicable to rotary bioreactor	http://www.synthecon.com/
Fibercell	FiberCell Systems Inc	Fiber bioreactor with hollow blood vessels mimicking anatomy facilitating perfusion of cell nutrients and waste removal	http://www.fibercellsystems.com/
MICA Microfluidic 3D Cell Culture	Cellasic	ECM protein gels integrated microfluidic perfusion plate platform for 3D culture	http://www.cellasic.com/
Quasi‐Vivo	Kirkstall	Specialized chamber for 3D culture system with multiplex perfusion capability in parallel or series configurations	http://www.kirkstall.com/
Human Organ‐on‐Chip	Emulate	Microengineered lung, liver, kidney, and intestine on chip	https://www.emulatebio.com/
RealBio D4 Culture System	RealBio Technology Inc.	Perfusion chamber with built‐in gas and media exchange compartments	http://www.realbiotechnology.com/
MC‐8	InvivoSciences, LLC	Specialized 3D cell culture chamber with capability development of tissue construct	http://www.invivosciences.com/
3DKube	Kiyatec	Perfusion flow circuit, segregated co‐culture, scaffold membrane with independent chamber	http://www.kiyatec.com/
Organoplate	Mimetas	High throughput Organ‐on‐Chip culture plate	https://mimetas.com/

Biochemical and morphological characterization can be done to confirm that these tissue layers resemble the original alveolar capillary interface.^[^ ^41^ ^]^ This LoC model mimics not only the lung‐specific differentiated morphology and functions but also more complex organ‐level functions that arise from interactions between multiple tissue types. Taking silica nanoparticles as a nanomaterial model, the LoC microdevices demonstrated cyclic mechanical strain to be the key to inflammatory responses and toxic potential shown in vivo with the whole mouse lung model.^[^ ^50^ ^]^ The nanoparticle transport across endo‐ and epithelial microvasculature has been shown as a function of mechanical strain applied on this microengineered device as one of the most complex integrated organ‐level functions that occur in the respiratory system.^[^ ^46^ ^]^ Because of the optical transparency of the device, it is possible to visualize and analyze the entire process with flexibility and its physiological response in real‐time at high resolution, which would be very challenging to do using traditional in vitro models or animal models. The in vivo anatomical hallmark of alveoli tight junction protein expression, TEER, and cyclic mechanical strain mimic in vivo conditions as a realistic model for regulatory toxicology research.^[^ ^51^ ^]^ These emerging technology models complex disease processes related to the inhalation pathway of the human body on in vitro platforms. In another three parallel channels LoC model, authors successfully model the cell‐matrix and cell–cell interactions with recapitulating vascular mechanical cues. In detail, physicochemical and biochemical analysis had shown unique results, when taking ZnO and TiO_2_ nanoparticles as model particle inhalation, which could not be possible using in vitro systems.^[^ ^52^ ^]^

## 5 Pros versus Cons of Advanced Organ‐on‐Chip Engineering Technology

Many structural and functional details have been included regarding three key aspects of human physiology on how Organ‐on‐Chip technologies can recreate function of organs, focusing on tissue barrier properties, parenchymal tissue function, and multi‐organ interactions.^[^ ^42^ ^]^ In the review by Zhang et al, specific systems are examined in terms of cell sources, functional hallmarks, available disease models, and future challenges like materials, cellular fidelity, multiplexing, sensing, scalability, and validation are outlined.^[^ ^42^ ^]^ However, organ on chip like other technologies is not without pros and cons as briefly described below in **Table** **3**.

**Table 3:** Pros versus cons of advanced Organ‐on‐Chip engineering technology

Pros	Cons
Organ‐on‐Chip devices can include dynamic environmental factors that improve cellular fidelity and compatibility with imaging.	Materials for Organ‐on‐Chip engineering need to be designed for both the engineering of the device and the support of cell culture.
Improvements to the expensive nature of the efficiency of drug discovery could create billions of dollars in savings for large companies.	Organ‐on‐Chip devices are difficult to scale to mass production.
Plastic membranes or microfluidic hydrogels can be functionalized into microfluidic channels to model human tissue's various barriers and interfaces.	Hydrogel's are, by nature, fragile which make it challenging to shape them into stable structures with a defined vascular–epithelial interface.
Separate microfluidic networks can be created by synthetic polymeric elastomer used as a scaffold for the development of vascularized functional tissues.	Organ‐on‐Chip devices need successful reproduction of biological functions. A high level of understanding is needed for industrial decision making and current validation protocols are limited.
To prompt the self‐fabrication of endothelial cells into perfusable microvascular network in hydrogels growth factors and biochemical signals are used.	Elastomers used in LoC devices are hydrophobic and very sticky to ENMs, growth factor, and protein supplements, which reduces their availability to cells.
Microfluidic cell culture device representing 13 organs was created to model inter‐organ interactions and quantify the relationship between organ volume and blood residence time.	Body‐on‐chop concept struggle with developing universal in vitro culture mediums for organ‐specific cells, functional aspects, and lack of organs specific multicellularity.
Using human iPSCs, epithelial and epithelial‐mesenchymal organoids have been developed like corneal and retinal tissue.	Organoids are challenged by reproducibility, diffusion, control over perfusion and input–output parameters, applicability of built‐in functional readouts.

## 6 Soft‐Robotics Integrated Breathing Lungs‐on‐Chip as Future Technology: Outlook and Future Directions

### 6.1 Limitations of Current Breathing Lungs‐on‐Chip and Opportunities Provided by Advancement in Soft‐Robotics

Organs on chip are sophisticated 3D‐microfluidic cell culture devices, which mimic complex in vivo mechanics and physiology on small chips.^[^ ^53^ ^]^ They can be attributed as BioMEMS devices with micro‐engineered organ or organ systems specific functions.^[^ ^54^ ^]^ The technologies in stretching and compression in 3D and cyclic strain devices are bridging the gap of biomimetic actuation of organs on chip devices, which needs cyclic beating (e.g., heart, lung, etc.). However, untethered or wireless actuation systems are missing in current devices. The micro manufacturing of these tiny organ chips is costly, needs expensive clean room facility for fabrication and involves massive expertise in manufacturing. Current Lab‐on‐Chip is designated as Lab‐around‐chip due to cumbersome pump fluidic device connections and external sensors sophistication needed to monitor the biological functions.^[^ ^55^ ^]^ Further limitations and disadvantages of these LoC technologies are referred to in Table 3 above. The human breathing LoC technology had a major impact and changed the way regulatory and funding agencies thought about in vitro testing. In the last decade, it has become one of the most important reasons why funding agencies created many new funding programs concerning Organ‐on‐Chip technologies such as placenta on chip, eye on chip, skin on chip, etc.^[^ ^56^ ^]^ In the US, major funding programs have been announced to support research activities that are focused on replacement of the animal models and developments of the micro physiological systems for preclinical trials. Moreover, there have been interdisciplinary collaboration projects where bioengineers, chemists, biologists, and clinicians synergistically work together. In the coming decades, there will be many opportunities in this very exciting research field.^[^ ^57^ ^]^

In the lungs, the immune response is composed of many different immune cells that are recruited from the blood rather than produced locally. The immune system plays an essential role in the immune response to foreign bodies, viruses, and particles, but it is largely neglected in most of these alternative models. Immune cells are vital to the immune response, but they are rarely discussed or mentioned in in vitro toxicology modules. Despite the complexity of these models, it is extremely difficult to reproduce in vitro a full lung inflammation process. So animal models, despite the ethical question, remain among the best methods for studying human lung function. When cultures of cells and tissues are used in inhalation toxicology, immune components must be incorporated to achieve physiologically equivalent conditions, for instance, by activating chemokines and cytokines. To reliably mirror in vivo models during in vitro experiments and draw significant extrapolation, we need to consider how to create the immune cell interactions in integrated lung models establishing adaptive and innate immune coculture systems with lung cells.

An exciting future use of LoC devices in regulatory toxicology takes advantage of the computational model to predict optimal doses that putatively cause lung injury. At the same time, it can be applied to in vitro system for specific chemical toxicants; an approach can be called as “reverse dosimetry” prediction utilizing Organ‐on‐Chip devices.^[^ ^58^ ^]^ They can be leveraged in conjunction with the in vivo physiologically based pharmacokinetic model to determine the resident times for toxicant compounds in each organ compartment and the optimal doses for treatment. It could also determine the relevant in vivo pharmacokinetic parameters such as mean Lethal Dose (LD50) or half‐maximal inhibitory concentration (IC50).^[^ ^59^ ^]^

### 6.2 Outlook and Future Prospect: Soft Stimuli‐Responsive Materials for Miniaturized Organ Development

Photoresponsive liquid crystal gels (LCGs) have enabled the design of light‐fueled soft‐robots, which can be remotely actuated and controlled wirelessly to move underwater. The LCGs provide local deformation upon shining the light enabling the soft robots to perform complex bending, crawling, jumping, and swimming underwater.^[^ ^60^ ^]^ The functions of the stimuli‐responsive materials can be adapted into perforated membrane formats like in Transwell where lung alveolar cells are co‐cultured with endothelial cells. The LCGs can be potentially used as a cell culture template to mimic the in vivo organ level breathing functions with alveolar cell monolayer cultured on the soft‐transparent LCGs membrane.

**Figure 8:**
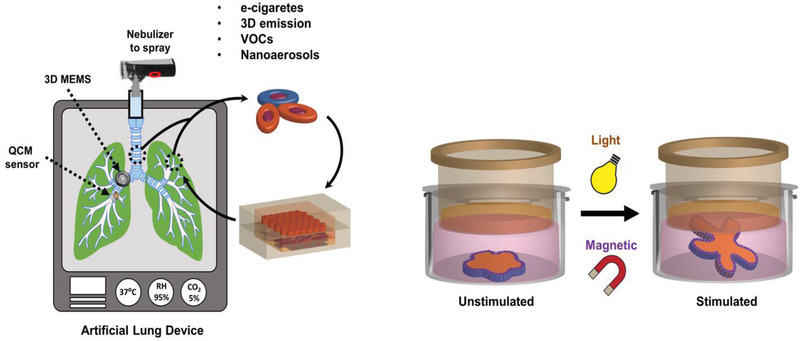
Tabletop Portable artificial lung device as futurist inhalation toxicity measurement device. The left panel is conceptually 3D printed soft elastomers. Mimicking the intricate similarity of different respiratory regions can be created with plug in capabilities of 3D BioMEMS devices harboring specific cells from thoracic, tracheobronchial, and alveoli specific, and mechanical stretching of an alveolar‐capillary interface via untethered actuation inspired by advances in soft robotics (right panel). The device has capability to integrate nanoaerosol production to expose the MEMS device neighboring QCM for real‐time dosimetry modeling with mist/cloud of testing substances (e.g., volatile organic compounds or VOCs, pollutant, e‐liquids aerosol, 3D emission from household printers, Nanomaterials exposure, etc.). It will combine both biological components and accurate exposure together for correct inhalation toxicity measurement.

The soft‐robotics advances have offered multiple degrees of freedom regarding the motions of these tiny soft‐robots, which could potentially provide advantages to the Lungs‐on‐Chip device improvement and throughput in inhalation toxicology.^[^ ^61^, ^62^ ^]^ Untethered versatile actuations mimicking the in vivo periodic upward and downward motion of the diaphragm during the inhale and exhale of the breathing cycle can be integrated in breathing Lungs‐on‐Chip devices. Researchers have designed multifunctional jellyfish‐like soft‐robots, which can achieve diverse controlled fluidic flows that generate extremely complex motions. For such a complex periodic motion and control, on chip format can be recapitulated incorporating soft materials (e.g., PDMS) containing magnetic particle as thin films. As shown in **Figure** **8**, the motion control can be performed by external electromagnetic coil setup, which can develop microengineered lung functions at the alveolar level.^[^ ^63^ ^]^ Such untethered magnetic actuation can lead to some advantages such as bimodal and bidirectional motions mimicking dynamics of lungs (inhalation/exhalation or diaphragm contraction/relaxation), compared to conventional breathing Lungs‐on‐Chip technology that depend on the applied pressure (e.g., pneumatic, hydraulic, etc.) creating unidirectional motion.^[^ ^64^ ^]^ In fact, these advances could be put together into tabletop format of 3D bioprinted lung with selective tuning the printed lung region to untethered magnetic or optical actuation as shown above in Figure 8. Such tabletop lung models could be very useful for real‐time exposure to variety of toxicants and their quantitative dose measurements. The important properties of soft actuators that equivocate the biological function for designing microengineered organs‐on‐chip are summarized in **Table** **4**.

**Table 4:** Summary of important factors for a soft actuator as in vitro lung. The remarks provide a framework for the material designs advancing surrogate lung models for the inhalation toxicology

Property	Remarks
Actuation strain and force production	Higher than 20% linear strain and 1–20 kPa shear stress created by the blood, surface tension, and interstitial flow at ALI with inflation pressure (about 5 cm H_2_O) and abdomen mechanical pulsations^[^ ^65^, ^66^ ^]^
Size scale	Organ‐to‐the microscale level^[^ ^67^ ^]^
Resistance to other stimuli	Mimicking barrier functions^[^ ^68^ ^]^
Power consumption	Mechanical ventilation‐induced power^[^ ^69^ ^]^
Actuation speed and response time	Mechanical impedance forced oscillation technique typically less than 10 Hz^[^ ^70^ ^]^
Biocompatibility and biodegradability	Low toxicity resorbable polymers as choice
Multifunctionality	Mechanotransducive and stimuli‐responsive^[^ ^71^ ^]^
Operating environment (in solution, air, etc.)	Both liquid (in vitro cell culture condition) and air (testing inhalation in cloud chamber)
Remote or tethered power source	Tethered pneumatic or untethered opto‐magnetic actuation^[^ ^72^ ^]^
Yield stress	Surface films of lung extract about 3 dyn cm^−2[^ ^73^ ^]^
Durability and fatigue resistance	Fatigue resistance biopolymeric materials^[^ ^74^ ^]^
Material viscoelasticity and Hysteresis	Soft elastomers^[^ ^75^ ^]^
Isotropic vs Anisotropy	Isotropic inflation^[^ ^76^ ^]^

In soft biomaterial‐inspired design, the piezoelectric materials have been applied to perforated membranes that could act as a micro‐pump to applied stress.^[^ ^77^ ^]^ Such actuation would allow the periodic closing/opening of pores leading to delivery of aerosol on cultured format. In conventional quartz crystal microbalance (QCM), the base nebulizer with the vibrating mesh acts as micro pump, which breaks liquids into micro/nano droplets (e.g., fine mist) to be delivered to the lungs. Moreover, there have been fundamental improvements to the ALI concept to bring closer resemblance of inhalation exposure in commercial ALI set up such as cloud chamber^[^ ^78^ ^]^ and P.R.I.T. ExpoCube.^[^ ^79^ ^]^

They provide various exposure scenarios such as online dosing monitoring, homogenous deposition, and versatile outcome and endpoint parameters. However, these systems lack actual in vivo lung‐like dynamicity, where soft robotics could be an alternative to conjugate dynamicity to the systems.^[^ ^62^ ^]^ Soft robotics could be designed to match viscoelasticity of multicellular systems that could play a crucial role in cell‐to‐cell communications as observed in ‘hydrogel’ like in vivo extracellular matrices.^[^ ^80^ ^]^ In addition, Artificial intelligence (AI) can assist in finding a match for toxicity predictions using suitable machine learning (ML) algorithm.^[^ ^50^ ^]^

**Figure 9:**
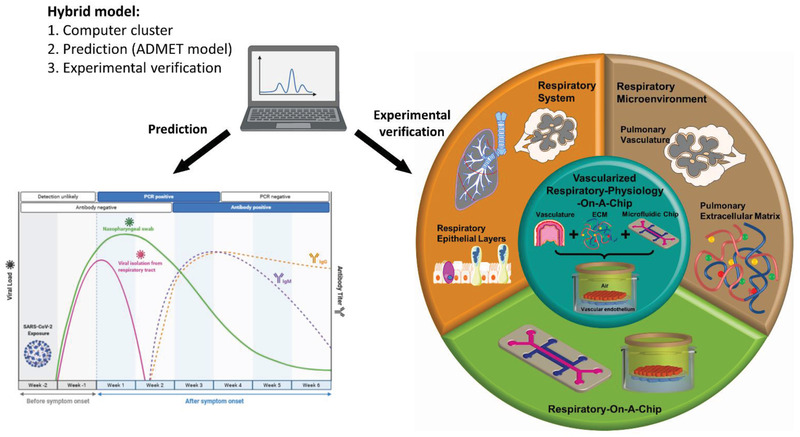
A hybrid multimodal system toxicology approach linking advances in ML and AI with in vitro Organ‐on‐Chip models. AI can boost vascularized respiratory physiology via computational prediction of testing of unknown toxicant and infectious agents. As an example, the immune profile variation can be predicted with computation model by training the data with known virus‐immune interaction databases after exposure to an unknown virus to cell on lung‐like in vitro platform. This may be empirically confirmed by adding a hierarchy of in vitro physiology improvements on a chip device to cut down the experimental range minimizing the time and cost of assay. Created with Adobe Illustrator and Biorender.

There is a huge need to build inhalation systems toxicology by combining hardware and software with advanced in cellulo/in vitro models based on AI integrated experimental methods.^[^ ^81^ ^]^ AI‐based applications in pulmonary medicine can improve to understand the vascularized respiratory physiology changes during tests when the inhalation pathway is exposed to unknown toxicants and infectious agents by using computational predictions via comparing databases that have the previous toxicants and infectious agents data on record. As shown in **Figure** **9**, current developments in respiratory Physiology‐on‐Chip allow the personalization of experimental toxicology models based on the modification of the inhalation exposure to cells under specified conditions. However, rapid improvement over the years is achieved by creating facile cell‐ALI in vitro. Recently, novel methods of immortalizing human alveolar epithelial lung cells using non‐viral vectors have been proposed for recapitulating the phenotype, functional and molecular characteristics of the healthy alveolar epithelium. It has been shown that immortalized cells exhibited AT1‐like characteristics as evidenced by low levels of alkaline phosphatase, receptor for advanced glycation end‐products (RAGE), podoplanin, and caveolin‐1 expression.^[^ ^82^ ^]^ This personalized experimental design addressing a specific toxicology question might be well qualified for acute inhalation testing. The next step of toxicity evaluation of mechanistic studies of inhalation research needs more complex systems to resemble in vivo conditions.

## Conflict of Interest

The authors declare no conflict of interest.

## Author Contributions

Conceptualization, A.V.S., P.L., and B.P.; data curation, A.V.S., A.R., and B.P.; writing—original draft preparation, A.V.S., S.W., A.R., and B.P.; writing—review and editing, A.V.S., C.S., B.P., P.K., S.B., and S.P.D.; graphic design and visualization, A.VS, B.P., and L.L.; supervision, A.V.S., P.L., and A.L.; project administration, A.V.S. and S.W.; funding acquisition, A.L. All authors have read and agreed to the published version of the manuscript.
